# Isolation, characterization and response surface method optimization of cellulose from hybridized agricultural wastes

**DOI:** 10.1038/s41598-024-65229-4

**Published:** 2024-06-21

**Authors:** Hauwa A. Rasheed, Adekunle A. Adeleke, Petrus Nzerem, Adebayo I. Olosho, Temitayo S. Ogedengbe, Seun Jesuloluwa

**Affiliations:** 1 Department of Industrial Chemistry, Nile University of Nigeria, Plot 681, Cadastral Zone C, Airport Road, Jabi, Abuja Federal Capital Territory, Nigeria; 2 Department of Mechanical Engineering, Nile University of Nigeria, Plot 681, Cadastral Zone C, Airport Road, Jabi, Abuja Federal Capital Territory, Nigeria; 3 Department of Petroleum and Gas Engineering, Nile University of Nigeria, Plot 681, Cadastral Zone C, Airport Road, Jabi, Abuja Federal Capital Territory, Nigeria; 4 Department of Chemistry, African University of Science and Technology, Abuja, Nigeria

**Keywords:** Cellulose, Agricultural waste, Surface response methodology, Alkaline treatment, Hydrogen peroxide bleaching, Chemistry, Materials science

## Abstract

This study explores the utilization of eight readily available agricultural waste varieties in Nigeria—sugarcane bagasse, corn husk, corn cob, wheat husk, melina, acacia, mahogany, and ironwood sawdust—as potential sources of cellulose. Gravimetric analysis was employed to assess the cellulose content of these wastes, following which two selected wastes were combined based on their cellulose content and abundance to serve as the raw material for the extraction process. Response Surface Methodology, including Box-Behnken design, was applied to enhance control over variables, establish an optimal starting point, and determine the most favorable reaction conditions. The cellulose extracted under various conditions was comprehensively examined for content, structure, extent of crystallinity, and morphological properties. Characterization techniques such as X-ray Diffraction, Scanning Electron Microscopy, and Fourier Transform Infrared Spectroscopy were employed for detailed analysis. Compositional analysis revealed sugarcane bagasse and corn cob to possess the highest cellulose content, at 41 ± 0.41% and 40 ± 0.32% respectively, with FTIR analysis confirming relatively low C=C bond intensity in these samples. RSM optimization indicated a potential 46% isolated yield from a hybrid composition of sugarcane bagasse and corn cob at NaOH concentration of 2%, temperature of 45 °C, and 10 ml of 38% H_2_O_2_. However, FTIR analyses revealed the persistence of non-cellulosic materials in this sample. Further analysis demonstrated that cellulose isolated at NaOH concentration of 10%, temperature of 70 °C, and 20 ml of 38% H_2_O_2_ was of high purity, with a yield of 42%. Numerical optimization within this extraction condition range predicted a yield of 45.6% at NaOH concentration of 5%, temperature of 45 °C, and 20 ml of 38% H_2_O_2_. Model validation confirmed an actual yield of 43.9% at this condition, aligning closely with the predicted value. These findings underscore the significant potential of combinning and utilizing agricultural wastes as a valuable source of cellulose, paving the way for sustainable and resource-efficient practices in various industrial applications.

## Introduction

The surge in agricultural commodities production and farming activities worldwide has significantly contributed to expanding the global food supply. However, a consequential outcome of this productivity is the accumulation of agricultural waste, generated during both production and processing stages. Agronomists categorize agricultural waste as unwanted materials produced in agricultural operations, often with minimal economic value compared to the cost of collection, transportation, and processing for reuse^1,2^. Remarkably, nearly all agricultural activities yield waste, with global annual waste accumulation projected to exceed 7.5 billion tons^3^. In Nigeria alone, this annual accumulation surpasses 32 million tons, with projections indicating a tripling of this figure due to escalating agricultural activities propelled by population growth^3^. Unfortunately, limited awareness of the benefits of recycling agricultural waste and its potential economic impact has led to widespread improper disposal and burning practices in many developing nations, posing environmental and infrastructural hazards. Nigeria, however, stands poised for significant waste-to-wealth opportunities, offering potential benefits across various sectors^4^. Leveraging these waste resources aligns with Sustainable Development Goals (SDGs) 9 and 11, focusing on infrastructure, industry, innovation, and sustainable urban development^5^.

Agricultural wastes are increasingly recognized as valuable feedstock for the production of sustainable base chemicals, particularly from the lignocellulosic components of plants^6,7^. Among the most promising and abundant cellulosic feedstocks derived from plant residues are corn stover, sugarcane bagasse, rice, and wheat straws/husk^6,8^, owing to their abundance, low cost, and non-food nature. With global production figures reaching 750 million metric tonnes for wheat, 1.2 billion for corn, 1.9 billion for sugarcane, and 500 million for rice annually^4,9,10^ the lignocellulosic components, mainly in the form of stover and bagasse, represent a vast source of cellulose^10^. In addition to these conventional feedstocks, sawdust emerges as another sustainable source of cellulose. Sawdust, or wood dust, is a byproduct generated on a large scale by wood industries, consisting primarily of cellulose (45–50%), lignin, and hemicellulose (20–30%)^11^. Annually, the USA alone produces 3 million tons of sawdust, much of which is disposed of in landfills^12,13^. However, open disposal practices pose significant health and environmental risks, particularly in developing countries like Nigeria, where they contribute to pollution and ecological threats^14,15^. Traditionally, sawdust finds various low-value applications such as charcoal preparation, absorbents for nitroglycerin or effluents containing heavy metals, filler in plastics, wood composts, and in linoleum and paperboard production^13,16^. Yet, challenges such as flammability and associated hazards limit its utilization, hindering its potential^7^. Therefore, exploring high-value products from sawdust represents a crucial step towards advancing the concept of biorefinery. Utilizing agricultural waste, including sawdust, as a source of cellulose offers both environmental and economic benefits^15^. By repurposing these wastes, industries reduce reliance on virgin materials, mitigating deforestation and lowering carbon emissions. Furthermore, it creates opportunities for revenue generation and job creation within the bioeconomy sector.

The initial phase of waste valorization involves fractionating agricultural waste into cellulose, hemicellulose, and lignin to enhance economic value and minimize environmental impacts. Pretreatment is essential to isolate these components by optimizing biomass surface area, reducing inhibitory compounds, and disrupting bonds^17^. Despite advancements, challenges persist, leading to contaminants in cellulose, hemicellulose, and lignin^18^. Effective pretreatment methods, such as thermochemical treatments using various chemicals and techniques such as microwave assisted chemical treatment are now emplyed to maximize yield and purity^19^. Recent research has demonstrated that deep eutectic solvents (DES) and ionic liquids (IL) are ecologically friendly solvents that have a positive impact on the purification, dissolving, and fractionation processes that turn lignocellulosics into biopolymers. Even so, the bulk of these processes concentrate on producing glucose so that it can be fermented to yield ethyl levulinate, bioethanol, and levulinic acid^20,21^. DESs are mixtures of pure compounds whose eutectic point temperature is significantly lower than that of the ideal mixture, whereas ILs are liquids made of salts with low melting points, usually less than 100 °C^21^. Combining a hydrogen bond donor molecule with a salt that accepts hydrogen bonds is a typical kind of DES^3^. Due to their high design flexibility, coordination power, catalytic activity, and tuneable solvent polarity, DESs and ILs are both considered designer solvents in organic synthesis^21^. When compared to organic solvents that are frequently used, ILs and DESs had lower volatility, which led to their initial classification as "green" solvents. However, more research revealed that the toxicity spectrum of these solvents is wide because of their chemical structures. Both DESs and ILs, particularly DESs, may have resource-intensive syntheses^22^. In addition, the cost of producing ILs is typically higher than that of industrial molecular solvents. Furthermore, microwave power has been explored as a potential pre-treatment method to improve different lignocellulosic by-products. There are numerous studies concerned with the extraction of cellulose and nanocellulose using microwave-assisted extraction such as feasibilty study of cellulose isolated from rice husk^23^, extraction of lignin from triticale straw, cellulose extraction from eucalyptus and pine tree wood waste^24^. It is important to note that even though cellulose extraction from biomass using wide range of organic solvents and ILs are termed environmentally benign, alkaline extraction still remain the most favorable. This is because its cheaper, low temperature and pressure are employed as well as fewer cellulose degradation, especially for low lignin content biomass such as herbacious crops and agriculrural residues like corn cob, wheat husk and sugarcane bagasse^21^.

Previous studies on isolating cellulose from agricultural waste have primarily concentrated on extracting it from a single agricultural waste stream^17,18,25–30^. Melesse et al.^27^ extracted cellulose from sugarcane bagasse through a convenient five-step treatment whereby NaOH concentration as well as reaction time were taken as variables and were optimized perfectly. Razali et al.^29^ in their recent study isolated cellulose from rice straw waste which was later modified to be used as a filler in structural composite application. Similarly, Feleke ta al.^26^ extracted cellulose from linseed straw through optimizing the retting time and multi-step alkaline-peroxide process using the Taguchi design of experiment. In addition to extracting cellulose from single waste stream, limited number of investigations have been carried out to optimize the extraction process. In light of these considerations, this study aims to isolate, optimize, and characterize cellulose from hybridized agricultural waste, a novel approach encompassing corn cob and sugarcane bagasse as agricultural wastes readily available in Nigeria. Employing Response Surface Methodology (RSM) including Box-behnken design (BBD) enhances control over variables, offering insights into optimal conditions for cellulose extraction. BBD is a second order design that offers design economy and precise prediction variance. Comprehensive characterization utilizing X-ray diffraction (XRD), scanning electron microscopy (SEM), and Fourier transform infrared spectroscopy (FTIR) will elucidate cellulose properties under various reaction conditions. The cellulose that is acquired will further be utilised as a feedstock for the synthesis of carboxymethyl cellulose, which may be employed as an additive in the two most widely used procedures in the oil and gas industry i.e. hydraulic fracturing and drilling.

## Materials and methods

### Materials

All chemicals used in this study were of analytical grade. Sodium hydroxide (NaOH) pellets were obtained from Molychem Mumbai, India. Hydrogen peroxide (38%) was purchased from Merck (Darmstadt, Germany). Acetone (97%) and ethanol (95%) were obtained from Sigma-Aldrich, Burlington MA. Glacial acetic acid (90%) was obtained from Amichem Research Lab, Dehradun, India. Citric acid from Kermel, Tianjin, China. The four types of sawdust used in this study were collected from the Suleja Central Market in Niger State. Sugarcane bagasse was obtained from Cooperate Juices, Sahad Stores, Garki Area 11, Abuja. Corn cobs and corn husks were gathered around the Galadimawa roundabout, FCT, Abuja, Nigeria. Wheat husk was obtained from a local market in Borno, Maiduguri, Nigeria.

### Compositional analysis of the selected agricultural waste

**Figure 1:**
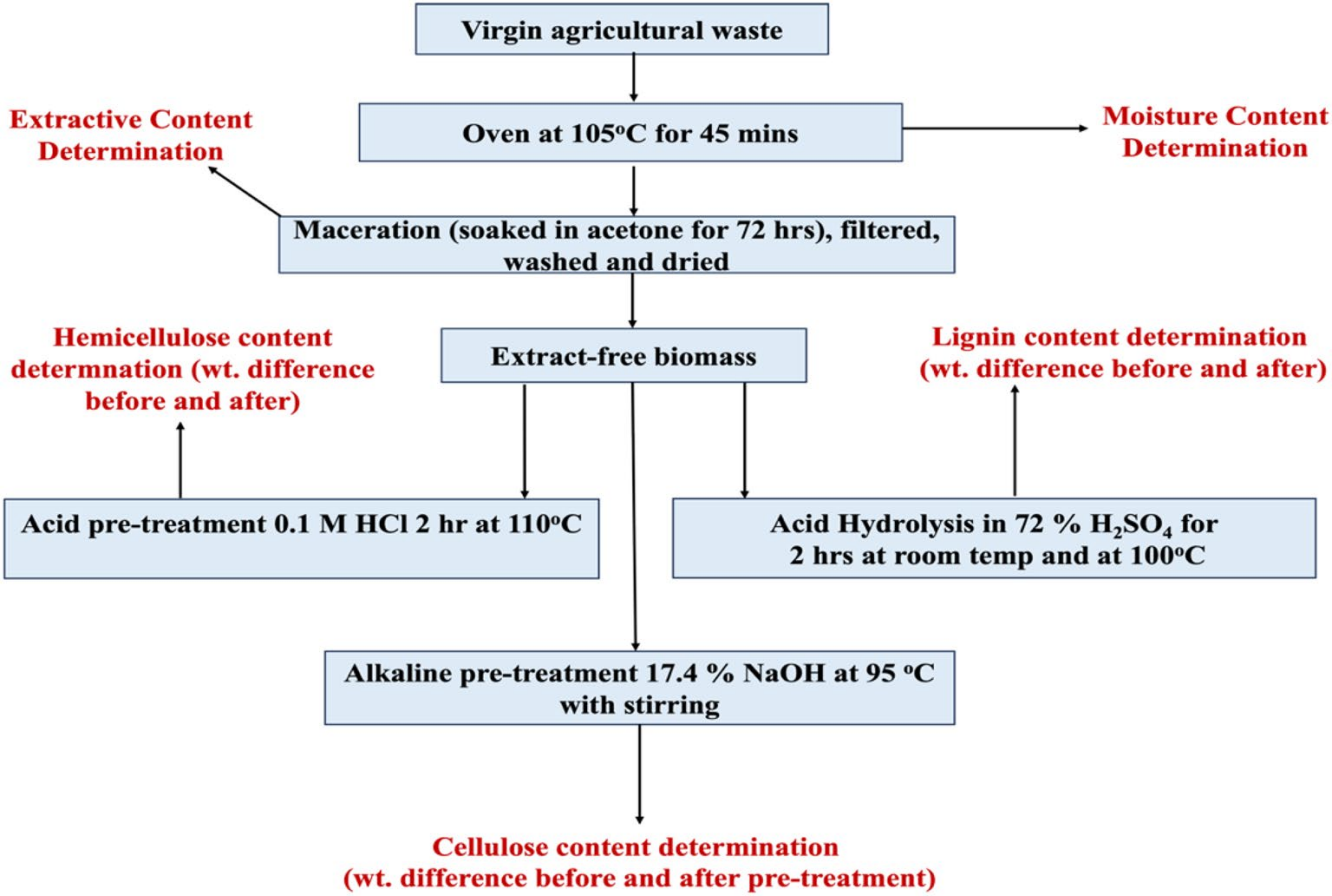
Flowchart of the chemical composition analysis of selected agricultural wastes.

To primarily quantify the percentage levels of cellulose, hemicellulose, lignin, extractives, and moisture, a compositional analysis (dry-weight basis) of the different biomass was conducted following TAPPI standard procedures^31^. A summary of these procedures is shown in Fig. 1. Acetone was employed as the maceration solvent (cold extraction). Before being macerated, every agricultural waste was carefully collected, cleaned, sun-dried, ground, sieved and placed in a Ziploc bag with the labels for later usage. For each sample, the percentage moisture, extractive, lignin, hemicellulose, and cellulose were calculated using the difference before and after every chemical treatment and drying^32–34^.

### Isolation of cellulose

**Figure 2:**
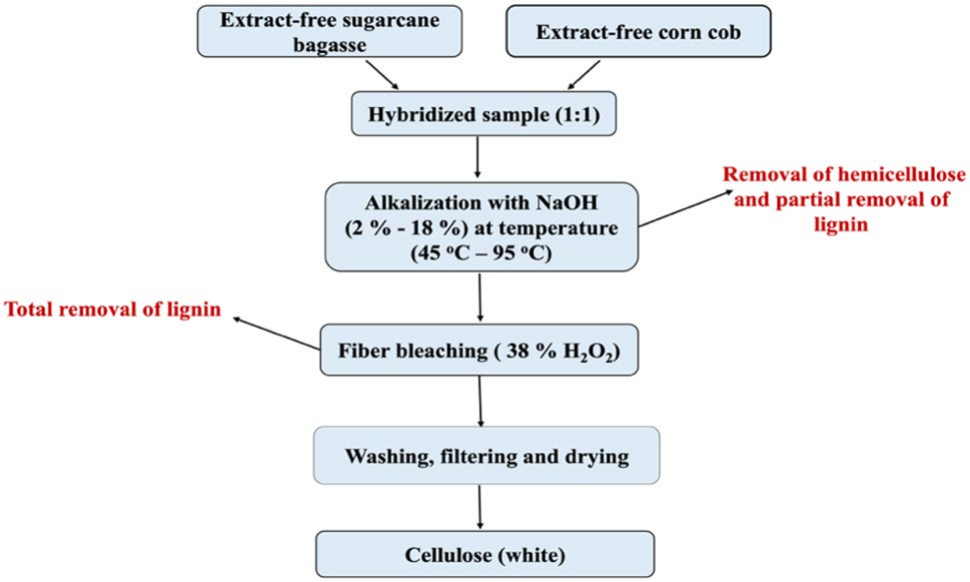
Flow chart of the synthesis of cellulose from a mixture of sugarcane bagasse and corn cob.

Cellulose was isolated from combined sugarcane bagasse and corn cob through sequential chemical treatment (Alkali treatment and hydrogen peroxide bleaching) described by Enriquez et al. and Fitriana et al.^25,35^ with slight modification. Instead of using glacial acetic acid to neutralize the mixture following the alkalization process, 0.2 M citric acid was employed in this instance. Corn cob and sugarcane bagasse were mixed with NaOH, diluted with distilled water, and filtered through Whatman No.4 filter paper. The resulting fibrous matter was then transferred to a clean beaker for bleaching, where 38% H_2_O_2_ was added, and the pH adjusted with 2 M citric acid. The mixture was then cooled, filtered, and dried before storage in a Ziploc container for future use. Figure 2 summarizes the cellulose extraction procedure. The % extraction yield of cellulose was calculated for each sample using Eq. (1).

1 $$\% cellulose \;yield = \frac{(weight \;of \;dried \;cellulose \;extracted) }{(weight \;of \;dried \;extract-free \;sample) }$$

### Experimental design for cellulose extraction

RSM which includes the BBD, was used with design expert software (version 13.0.5.0) to design the experiment to isolate cellulose from the combined sugarcane bagasse and corn cob waste. The NaOH concentration (A), extraction temperature (B), and volume of H_2_O_2_ (C), were the independent factors while the (dependent variable) response was % cellulose yield. The polynomial equation (Eq. 2) was employed to characterize the effects of variables, incorporating linear, quadratic, and cross-product terms.

2 $$Y=\beta 0+\sum_{i=1}^{k}\beta iXi+\sum_{i=1}^{k}\beta ijX{i}^{2}+\sum_{i<j}^{k}\sum_{j}^{k}\beta ijXiXj+\varepsilon$$

where, Y refers to yield of cellulose, $\beta$ is the regression coefficients, k is the number of factors studied in the experiment and $\varepsilon$ is and the residual error, respectively. The experimental factors were coded according to Eq. (3) for the development of the regression equation.

3 $$X=\frac{\left({X}_{i}-{X}_{0}\right)}{\Delta {X}_{i}}$$

where $i=\text{1,2},\dots ,k$. $X$ is the dimensionless value of an independent variable,${X}_{i}$ is the real value of an independent variable, ${X}_{0}$ is the real value of the independent variable at the center point, and $\Delta {X}_{i}$ is the step change value^36^. Table 1 presents the RSM design along with coded levels. These factors selected based one-factor-a-time preliminary optimization assessment. The estimation of the requisite number of experimental runs to develop the model is formulated as N = 2k(k − 1) + C_0_, where k denotes the number of factors, and C_0_ represents the count of central points^37^. This generated 17 experimental runs. To mitigate the influence of unforeseen variability in the observed responses, the experimental runs were randomized^38^.The significant terms (p-value < 0.05) of the model were determined through analysis of variance (ANOVA). The effectiveness of the model in predicting response values was assessed. The optimized conditions were confirmed through experimental validation under the optimized settings.

**Table 1:** Design experiment summary.

Independent variables	Symbols	Coded levels		
		−α	0	+ α
NaOH conc. (%)	A	2	10	18
Temperature (^o^C)	B	45	70	95
Volume of H_2_O_2_ (mL)	C	10	15	20

### FTIR analysis

The Agilent Cary 600 series FT-IR spectrometer was used to evaluate the characteristics of the functional groups of the produced cellulose samples as well as the untreated sample. The FTIR spectra were recorded in a transmittance range of 4000 cm^−1^ to 650 cm^−1^.

### SEM analysis

A Phenom ProX desktop scanning electron microscope with energy dispersive X-ray capability (SEM–EDX) was used to study the microstructure of both the synthesized cellulose and untreated samples. The samples were scanned at 15 kV at magnification of 1000×. Prior to analysis, the sample's surface had a tiny, conductive layer of gold applied to it.

### XRD analysis

The Rigaku miniflex x-ray diffractometer manufactured by Japanese X-ray scientific, analytical, and industrial instrumentation cooperation was used. The degree of crystallinity of the raw material and isolated cellulose was studied using an XRD diffractometer. The device was operated with Cu K alpha radiation at a wavelength of 1.54 angstroms at 40 kV and 15 mA. Crystallinity index (C.I) calculation used a Segal et al.^39^ proposed formulation. In this approach, the x-ray total crystallinity (%) of cellulose is calculated from the peak height ratio between the intensity of the crystalline peak (I_crystalline_) and total intensity after subtraction of background signal (I_amorphous_ + I_crystalline_) as shown in Eq. (4)^37^.

4 $$\text{C}.\text{I }(\text{\%}) =\frac{{I}_{crystalline}}{{I}_{amorphous }+ {I}_{crystalline}} \times 100$$

## Results and discussion

### Compositional analysis and FTIR characterization of the selected agricultural wastes

The chemical compositions of the eight agricultural wastes are summarized in Table 2. The experiment was conducted in duplicates. Cellulose was shown to be the predominant component in all biomass samples, surpassing both hemicellulose and lignin contents. These findings are consistent with established literature^27,34,40–43^. According to the results, sugarcane bagasse offered the largest percentage of cellulose (41 ± 0.41%) and the lowest amount of hemicellulose, making it an excellent choice for cellulose isolation. Corn cobs also had low lignin levels and a significant amount of cellulose (40 ± 0.32%). In addition to their respective cellulose contents, sugarcane and corn cob are readily available and require little preparation. Therefore, they were selected to form the raw materials for isolation. The average lignocellulose composition of whole corn cob reported by several researchers is in the range of 33 to 43% cellulose, 26 to 36% hemicellulose, and 17 to 21% lignin. Similarly, sugarcane bagasse contains 38 to 50% cellulose, 15 to 28% hemicellulose and 13 to 24% lignin^44^. The quantity of these components has a considerable impact on their accessibility and suitability for bioproducts and biopolymer synthesis. Given its abundant and cost-effective availability, agricultural waste in general stands as a promising source of cellulose, driving forward green economic growth.

**Table 2:** Chemical composition of the different agricultural wastes.

Lignocellulosic type	Cellulose (%)	Hemicellulose (%)	Lignin (%)	Moisture (%)	Extractives (%)	Others (%)
Sugarcane bagasse	41 ± 0.41	18 ± 0.89	16 ± 1.1	6 ± 0.76	6 ± 0.76	13
Corn husk	36 ± 0.71	24 ± 0.88	13 ± 0.23	5 ± 0.96	5 ± 0.31	17
Wheat husk	34 ± 0.87	18 ± 0.31	14 ± 0.34	5 ± 0.34	7 ± 0.71	22
Corn cob	40 ± 0.32	28 ± 0.21	12 ± 0.76	7 ± 0.53	3 ± 0.78	10
Mahogany sawdust	37 ± 0.67	24 ± 0.98	17 ± 0.98	9 ± 0.87	7 ± 0.66	7
Acacia sawdust	39 ± 0.91	18 ± 0.22	16 ± 0.87	8 ± 0.72	6 ± 0.43	13
Iron wood sawdust	38 ± 0.56	22 ± 0.12	15 ± 0.89	6 ± 0.81	6 ± 0.79	13
Melaina sawdust	39 ± 0.95	24 ± 0.54	9 ± 0.23	9 ± 0.05	5 ± 0.44	14

**Figure 3:**
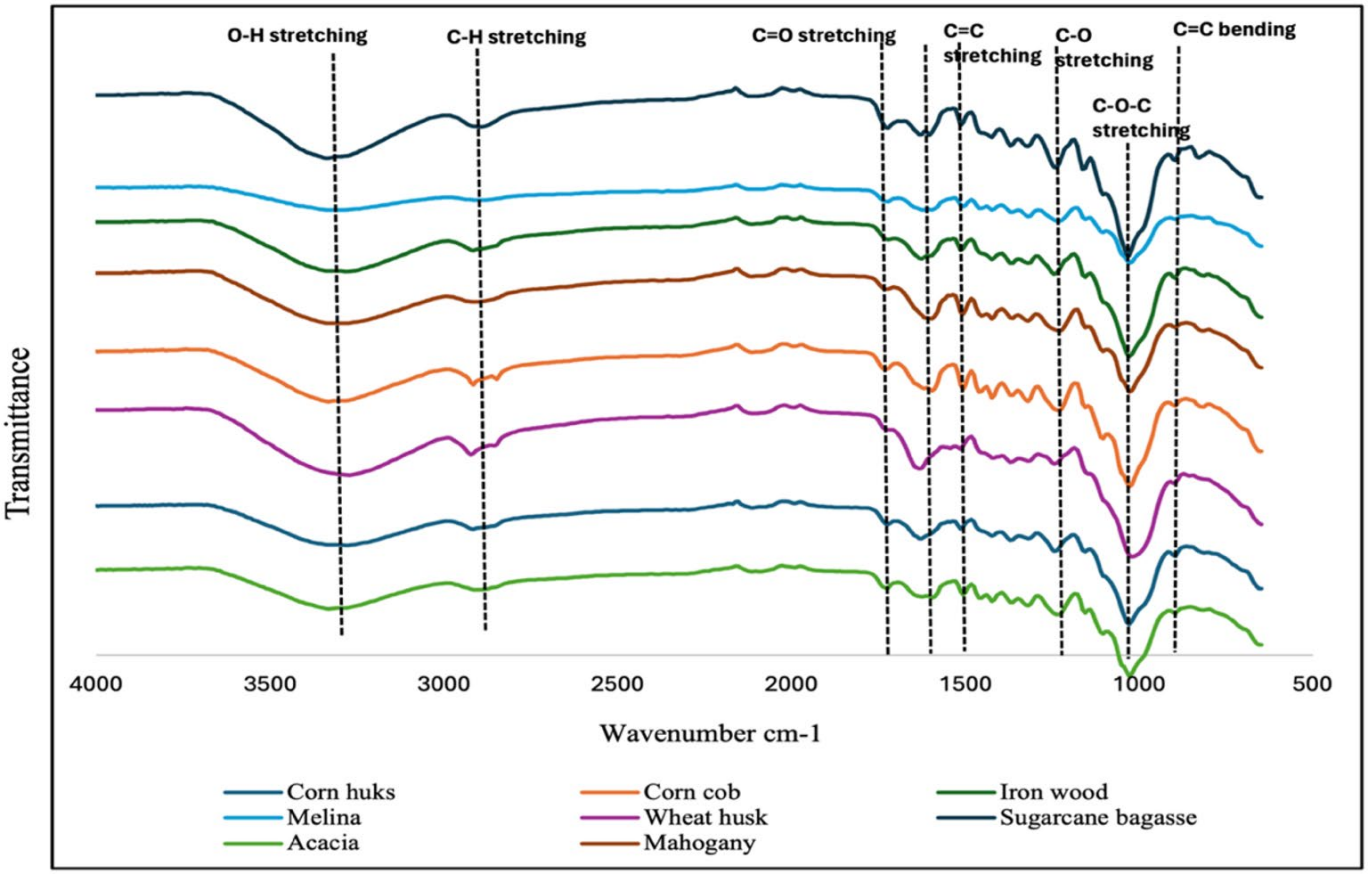
FTIR spectra of the eight agricultural wastes selected for compositional analysis.

Figure 3 shows the FTIR spectra while the polymers, allocated bands, and functional groups were identified in Table 5. Owing to the intricate nature of the eight biomass samples, their spectra displayed several peaks along with a range of functional groups, including aromatics, alkenes, and carbonyls. The strong correlation between the spectra and peaks indicates that the biomass material has similar functional groups. The O–H group of the intermolecular and intramolecular hydrogen bonds in the cellulose molecules exhibits free O–H stretching vibrations, which are the dominant broad band present in all spectra at about 3300 cm^−1^. The organic components of polysaccharides are typically linked to the C-H stretching vibration observed in the spectra between 2800 and 2900 cm^−1^. Similar findings on various plant biomass for cellulose extraction were reported in previous works^5,41,45,46^. The C=C aromatic skeletal vibration of hemicellulose and lignin in the structure has been linked to the FTIR spectra of the two materials, which are represented by peaks in the wavenumber at approximately 1500 and 1700 cm^−1^, respectively. An absorption band at around 1200 cm^−1^ is linked to the ether linkage's C–O stretching. The C–O–C of the pyranose skeletal ring is connected to the strong absorption peaks near the 1050 cm^−1^ linked explicitly to cellulose^27,33,41,47^. The results obtained from this investigation align with the conclusions documented in previous studies. Hence, these bands resemble those found in the FTIR spectra of lignin, hemicellulose, and cellulose. In comparison, the intensity of the OH peak in sugarcane bagasse is notably higher, while the intensity of the C=C stretching peak is lower. This suggests a relatively higher presence of cellulose and hemicellulose, aligning with the findings of the compositional analysis detailed in Table 2.

### Cellulose isolation and optimization

**Figure 4:**
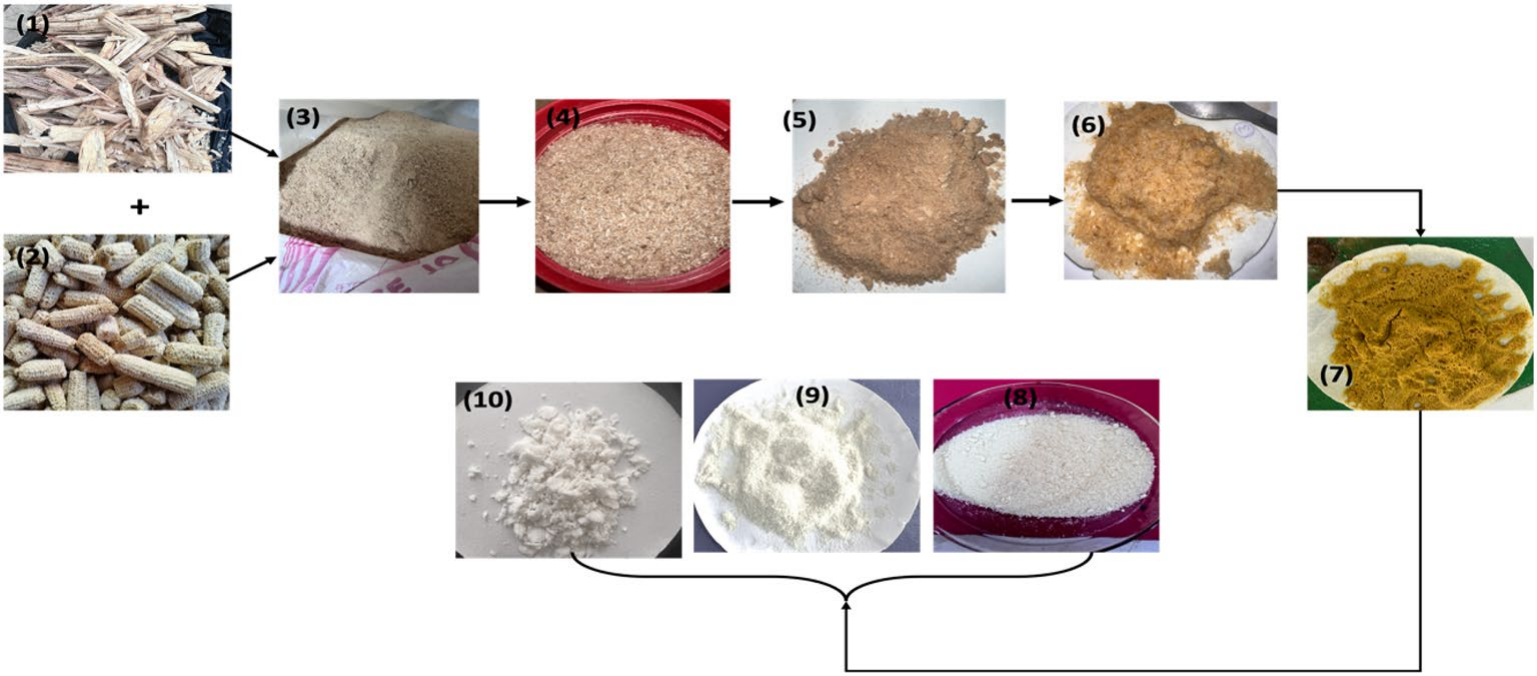
Photographs of (1) sugarcane bagasse, (2) corn cob, (3) pulverized hybridized sample, (4) and (5) sieved sample, (6) & (7) alkaline treated sample, (8), (9) & (10) isolated cellulose (bleached).

To efficiently isolate cellulose, biomass must undergo thorough pre-treatment to remove non-cellulosic components, thereby enriching the cellulose content and enhancing its accessibility during subsequent processing stages, while also facilitating the solubilization of lignin and hemicellulose^28,35,48,49^. Figure 4 illustrates some of the extracted cellulose samples, including those treated with alkali and those devoid of extractives. Notably, alkalization prior to bleaching had minimal impact on the colour of the isolated cellulose, with a transition to an off-white hue observed post-bleaching. This finding resonates with observations made by Enriquez et al.^35^ during the cellulose extraction process from spring-harvested switchgrass. Table 3 summarizes the percentage yield of cellulose obtained under various reaction conditions. Appreciative yield was achieved at 2% NaOH and 45 °C with 10 mL H_2_O_2_, while a low yield resulted from treatment with 18% NaOH at 95 °C with 20 mL H_2_O_2_. This discrepancy may stem from incomplete dissolution of lignin and hemicellulose by 2% NaOH at 45 °C, leaving behind non-cellulosic cellulose, or from overly harsh reaction conditions causing cellulose degradation, as evidenced by the lowest yield from treatment with 18% NaOH at 95 °C. While executing the procedure under ideal reaction conditions at its lowest possible level produced a higher percent yield, the resulting purity was not at its best. This further shows that, even if working under such ideal conditions could seem profitable, the isolated cellulose may still contain a desired proportion of non-cellulosic components when it comes to cellulose isolation from lignocellulosic. So, it is highly recommended to further define isolated cellulose to achieve the optimal reaction conditions and create a decent and pure product. Seven of the seventeen samples labelled A to G (Table 3) were chosen for characterization since they were analyzed under various reaction circumstances (high NaOH concentration/temperature as well as low NaOH concentration/temperature). This will help determine whether the alkalization reaction is greatly affected by temperature and what concentration is best for isolating the cellulose.

**Table 3:** Factors and response for process optimization.

Run	Selected samples for characterization	Factor 1 A: NaOH conc. (%)	Factor 2 B: Temperature (deg C)	Factor 3 C: Volume of H_2_O_2_ (mL)	Response: % yield
1		10	45	10	42
2		10	70	15	42
3	A	2	95	15	43
4		2	70	15	45
5		2	70	20	45
6	B	10	95	20	41
7	C	10	70	20	42
8		10	70	15	39
9		18	70	20	42
10		10	70	10	43
11	D	18	45	15	40
12	E	10	45	10	42
13		18	70	10	39
14		10	70	15	43
15	F	2	45	10	46
16	G	18	95	20	37
17		10	95	20	43

**Figure 5:**
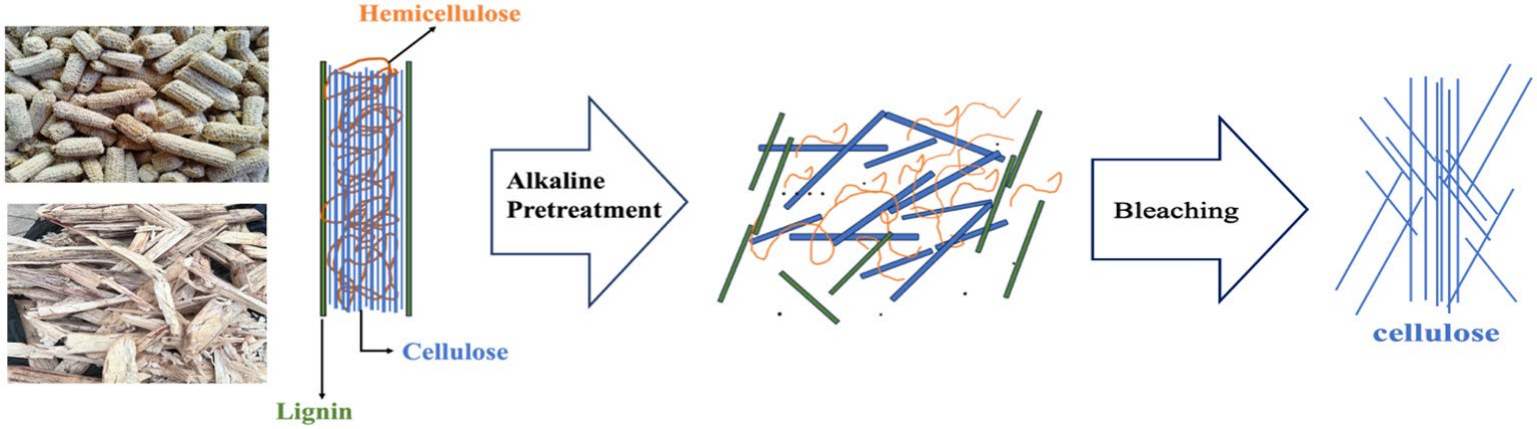
Schematic diagram of the two step alkaline treatment and hydrogen peroxide bleaching.

It’s worth mentioning that alkaline treatment disrupts the intricate matrix among cellulose, hemicellulose, and lignin present in lignocellulosic materials, facilitating cellulose isolation^28^. Exposed components are subsequently solubilized and washed away (Fig. 5), with alkali treatment effectively removing hemicellulose, waxes, oils, and a portion of lignin. The efficiency of impurity removal is influenced by temperature, duration, and alkali concentration. During treatment, cellulose undergoes division, rearrangement, and compaction to alleviate internal strain, resulting in increased hydroxyl group availability for chemical reactions with fibers and polymers. Moreover, alkali solutions disrupt hydrogen bonds and alter fiber arrangement, enlarging fibers and reducing crystallinity. Subsequent neutralization and drying processes modify porosity and elevate crystallinity, ultimately renewing cellulose fibers^35,50^.

The peroxide bleaching procedure, conducted under alkaline conditions, yields effects akin to alkali treatment while efficiently whitening natural fibers. In alkaline environments, H_2_O_2_ dissociates into hydrogen ions (H^+^) and perhydroxyl ions (OOH^.^). The perhydroxyl ion, a strong nucleophile, is the chemical species responsible for the bleaching effect on natural fibers. The release of H^+^ explains its behaviour as a weak acid, while the ^−^OOH interacts with another hydrogen peroxide molecule, forming free radicals. These ions (^**.**^OH and ^**.**^OOH) continue to react with unreacted hydrogen peroxide, decomposing it and generating perhydroxyl radicals until all hydrogen peroxide molecules are consumed, resulting in water and oxygen as the final products^50,51^. However, in the presence of pigments, electrons are abstracted from the chromogens, leading to their breakdown in a redox reaction, thereby reducing their optical absorption^50^. When used in conjunction with alkali treatment, the bleaching process can solubilize and remove remaining non-cellulosic components, particularly lignin, which may not have been effectively removed previously, while simultaneously providing a whitening effect^26^. Conspicuously, the effectiveness of hydrogen peroxide bleaching increases with its pH, which is why bleaching fibers after the alkali treatment is always preferable^25^.

**Figure 6:**
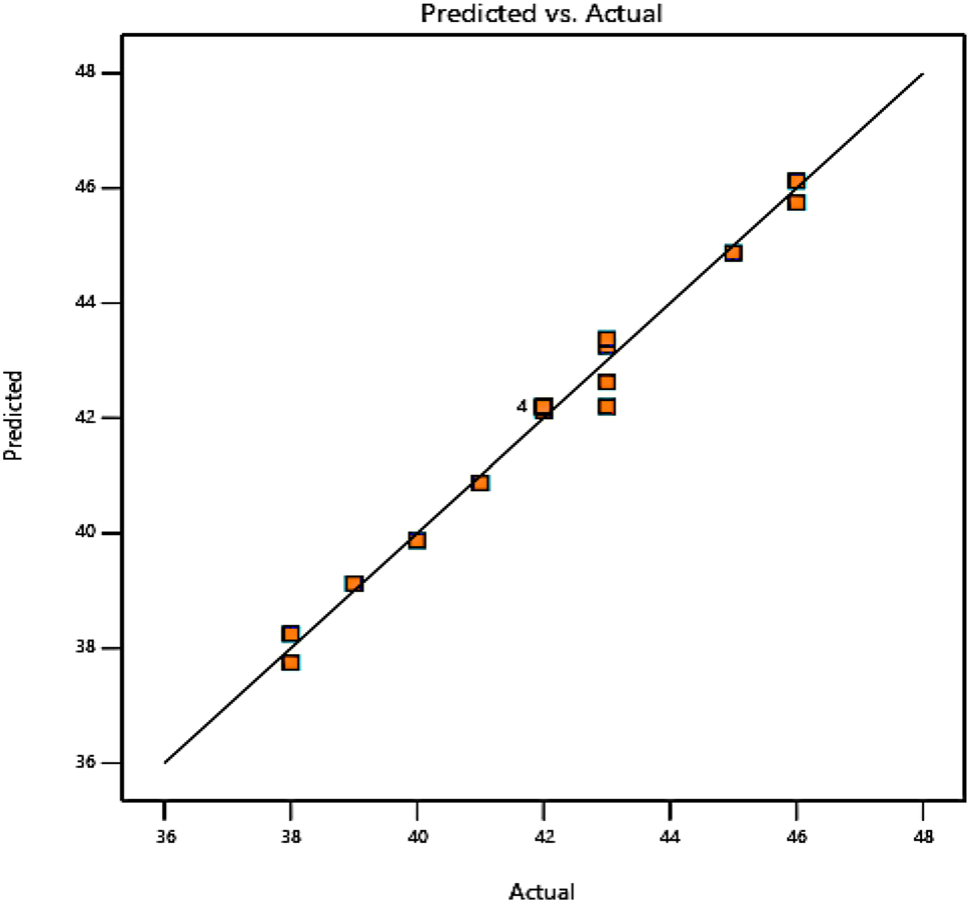
Plot of predicted vs actual of isolated cellulose yield.

Table 4 presents the analysis of variance (ANOVA) results of the quadratic model for cellulose isolation. The coefficient of determination (R^2^) for the quadratic model was 0.9843. In a normal way, a R^2^ value higher than 0.9 illustrates a high correlation which suggest that 98.43% of the total variance can be explained by the model^48^. Similar finding was reported when cellulose was extracted from jute fiber and the extraction process was optimized using BBD^35^. The adjusted R^2^ was reported to be 0.9582 which is high enough to confirm the significance of the model. The f-value of 37.69 and the extremely low probability value of 0.0001 indicate the significance of the model as well. With a lack of fit value of 0.3152 (not significant), the model was found to be sufficient to both predict the response and explain how variables affected it. Table 3 appropriate precision is demonstrated by the signal-to-noise ratio of 21.3639, which further suggests an adequate signal. As a result, this model can be utilized to explore the design space. Overall, the ANOVA findings confirm that the model is suitable and reliable for isolating cellulose. Figure 6 shows the plot of the response predicted from the empirical model and actual values obtained from the experiment. Most of the data points on this plot lie close to the experimental values due to closeness of the actual and predicted yields indicating good performance.

**Table 4:** ANOVA for quadratic model cellulose isolation.

Source	Sum of squares	d*f*	Mean square	f-value	p-value	
Model	101.00	9	8.95	37.69	0.0001	Significant
A-NaOH Conc	58.09	1	36.00	151.58	< 0.0001	Significant
B-temperature	3.62	1	2.00	8.42	0.0273	
C-Vol. of H_2_O_2_	0.0008	1	0.1250	0.5263	0.4955	
AB	0.9488	1	2.25	9.47	0.0217	
AC	1.83	1	1.000	4.21	0.0860	
BC	0.6542	1	2.25	9.47	0.0217	
A^2^	4.97	1	0.518	2.17	0.1910	
B^2^	0.0029	1	1.52	6.38	0.0449	
C^2^	1.36	1	1.78	7.49	0.0339	
AB^2^	0.125	1	0.1250	0.5263	0.4955	
Residual	1.42	6	0.2375			
Lack of fit	0.6250	2	0.3125	1.56	0.3152	Not significant
Pure error	0.8000	4	0.2000			

Fit statistics						
Std. dev.	Mean	C.V %	R^2^	Adjusted R^2^	Predicted R^2^	Adequate precision
0.4873	42.06	1.16	0.9843	0.9582	0.7114	21.364

**Figure 7:**
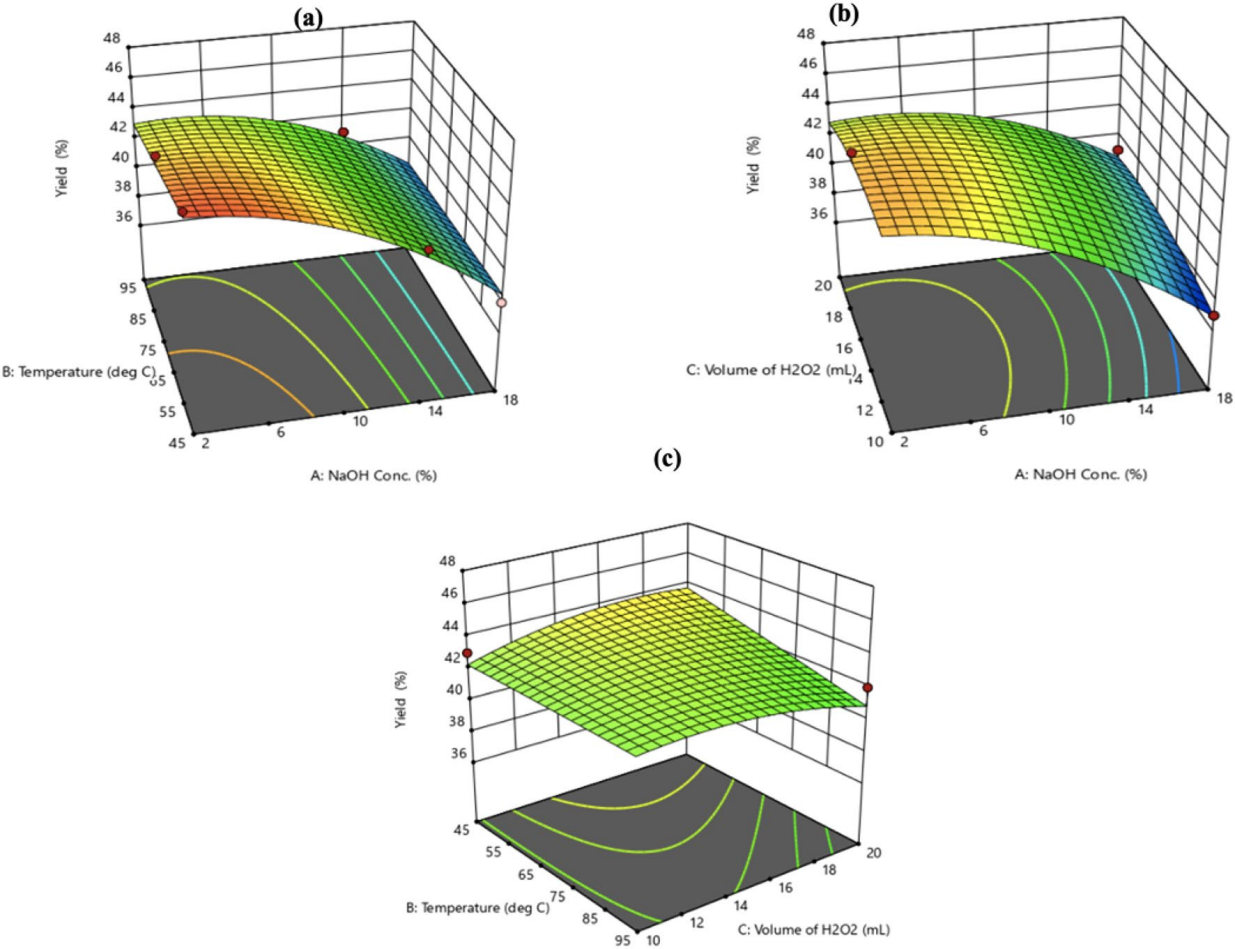
3D surface response of variables against % cellulose yield.

**Figure 8:**
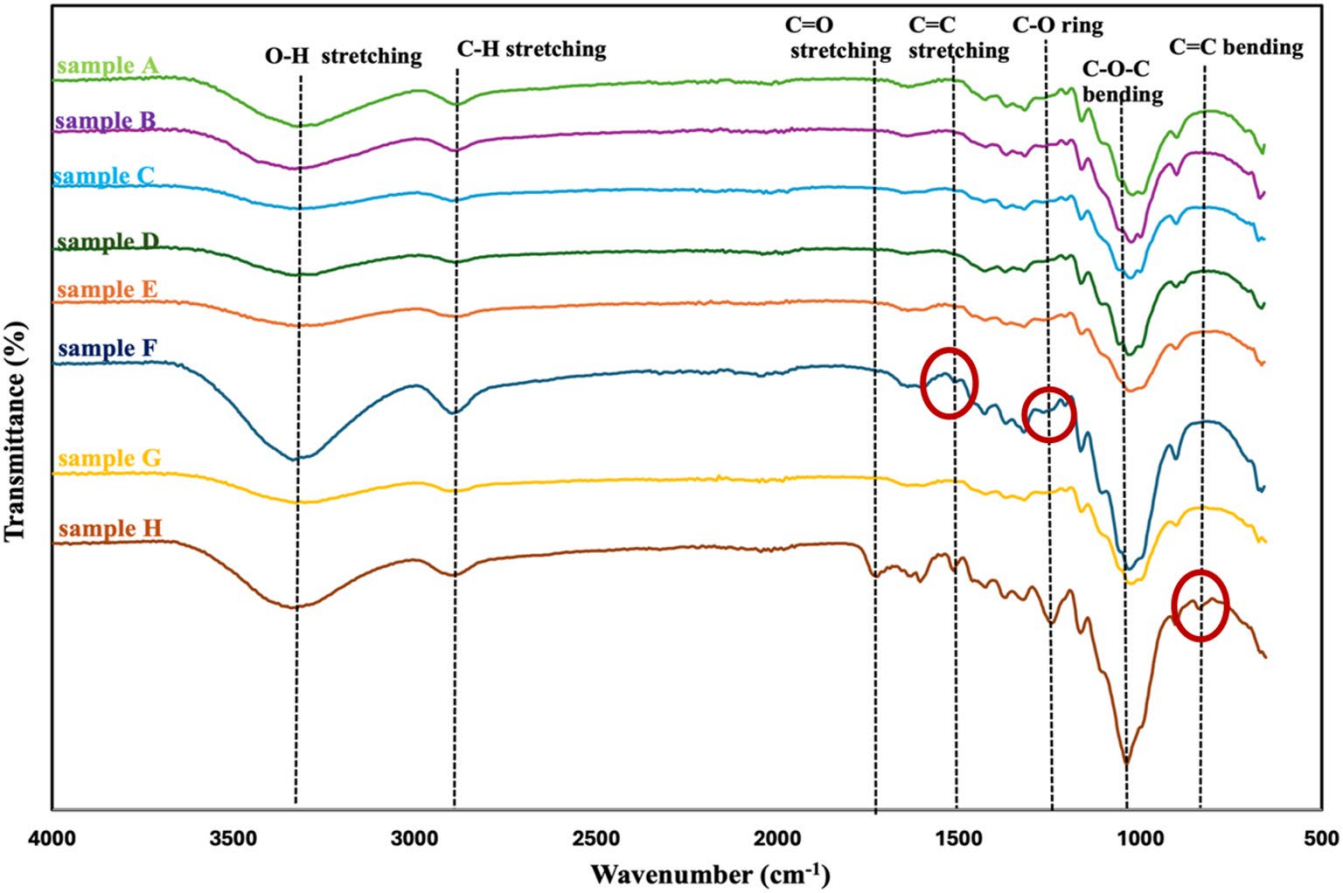
FTIR spectra of untreated sample and isolated cellulose (bleached).

Most of the data points on this plot lie close to the experimental values due to closeness of the actual and predicted yields indicating good performance. The maximum experimental yield achieved was 46% and it occurred when the variable factors were set to 2% NaOH, 45 °C and 10 mL. Figure 8 displays a 3-D response plot created from response surface approach that examines the impact of synthesis parameters on the cellulose isolation yield. Each 3-D plot represented combinations of the two test variables while the other variables were maintained at the center level. The relationship between the yield percentage from cellulose isolation and temperature (B), NaOH concentration (A), and their reciprocal interactions is depicted in Fig. 7a. Reduced temperature and extraction time for the cellulose isolated resulted in an increase in the yield that is achieved. This is because not enough NaOH was present to eliminate most of the hemicellulose and some fraction of the lignin. In Fig. 7b, the yield of isolated cellulose is used to illustrate the impacts of NaOH concentration (A), H_2_O_2_ volume (B), and their mutual interaction which collectively could be observed. Yet, the yield of cellulose increased with the reduction of NaOH, and the yield of cellulose increased with increasing volume of H_2_O_2_. The influence of temperature (B), H_2_O_2_ volume (C), and their reciprocal interactions with respect to the isolated cellulose yield are also displayed in Fig. 7c. It is noteworthy that these two factors did not interact excessively. However, the highest yield was noted at the lowest temperature and the highest possible H_2_O_2_ volume. Overall, the exhibited 3-D graph demonstrated the degree of interaction between the independent factors and the % cellulose yield.

### Structural analyses of isolated celluloses

FTIR analysis was conducted on selected cellulose yields (A–G) from Table 3, encompassing a spectrum of extraction conditions, to assess their composition in comparison to the extract-free sample H. The results are depicted in Fig. 8. The observed FTIR spectra exhibit significant similarity, indicating comparable chemical compositions as corroborated by compositional analysis results. The broad absorption bands evident in all spectra around 3300 cm^−1^ are attributed to the stretching vibration of inter-molecular and intra-molecular O–H groups, indicative of the presence of aliphatic moieties in polysaccharides^47^. Similarly, the C–H vibrations observed in the region of 2900 cm^−1^ across all spectra are associated with the general organic constituents of the major components, namely the alkyl group present in all three major components. Notably, a peak evident in the region of 1700 cm^−1^, related to the C=O stretching vibration found specifically in lignin, was observed solely in the spectrum of the raw sample (sample H), and was absent in the spectra of the isolated cellulose. This observation suggests the successful removal of these components following the alkali and bleaching processes. Additionally, peaks observed around 1500 cm^−1^ and 1300 cm^−1^ correspond to the C=C stretching vibration of lignin and C–O stretching vibrations of hemicellulose, respectively.

Furthermore, significant peaks observed at 1080 cm^−1^ and 890 cm^−1^ are associated with C–O–C and C–O stretching at the β-glycosidic links, consistent with the presence of cellulose in all samples. Importantly, the absence of the C=C bending vibration at approximately 830 cm^−1^, initially present in the spectrum of the untreated sample, suggests the successful elimination of lignin from the isolated cellulose following the two-step chemical treatment performed on all samples^47^. Among the samples, Sample F (2% NaOH, 45 °C, 10 mL H_2_O_2_) yielded the highest cellulose content based on compositional analysis (Table 3). However, the FTIR spectra revealed small peaks around 1500 cm^−1^ and 1200 cm^−1^, indicative of residual C=C stretching and C–O ring vibrations, respectively, suggesting incomplete removal of lignin. This indicates that the isolation of cellulose under mild reaction conditions may not be sufficient to completely solubilize all non-cellulosic components, resulting in impure cellulose. Conversely, Sample G (18% NaOH, 95 °C, 20 mL H_2_O_2_) exhibited the absence of peaks associated with hemicellulose and lignin, indicating successful elimination of these fractions despite yielding lower cellulose content. However, the severity of the reaction conditions may have led to cellulose degradation, resulting in decreased cellulose recovery. For the remaining samples, A, B, C, D, and E, their individual spectra demonstrate satisfactory removal of hemicellulose and lignin, as no peaks associated with these components were observed (Table 5).

**Table 5:** FTIR bands, functional groups and assigned polymer.

Bond type	Wavenumber (cm^−1^)	Assigned polymer
O–H stretching	3300	C, H, L
C–H stretching	2900	C, H, L
C=O stretching	1700	H, L
C=C stretching	1500	L
C–O (ring)	1230	L
C–O–C stretching of glycosidic link	1050	C, H, L
C=C bending	830	L

*C* cellulose, *H* hemicellulose, *L* lignin.

### SEM analysis

**Figure 9:**
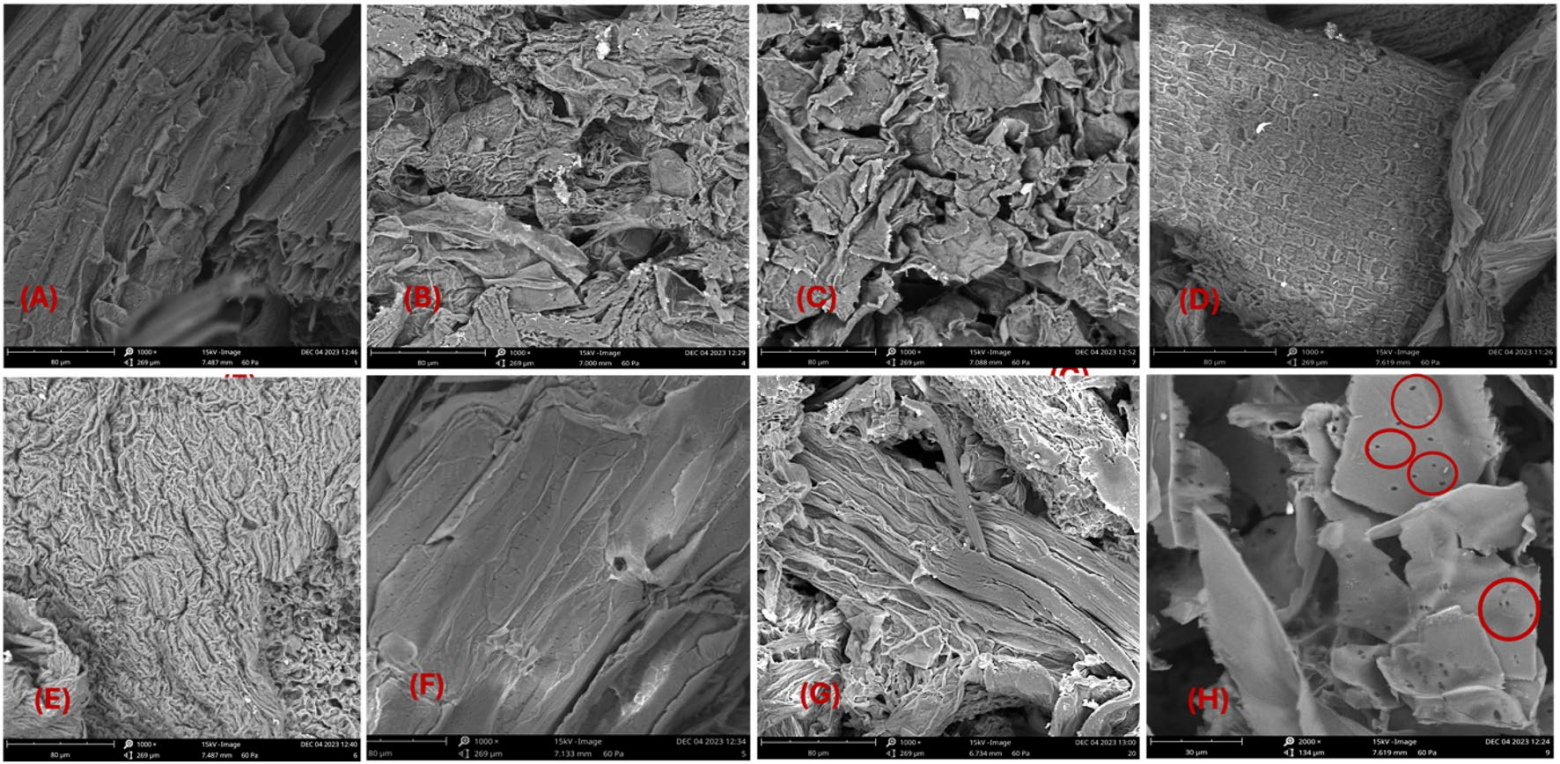
Micrograph of extract-free sample (**H**) and synthesized cellulose (**A** to **G**).

The SEM analysis in Fig. 9 reveals the surface morphology of the extract-free and the isolated cellulose samples across various stages. In its untreated state (Fig. 9H), the sample exhibited a smooth, pristine surface with scant pores, reflecting its composition comprising hemicellulose, pectin, wax, and other impurities, all bound together by lignin^35^. Following alkaline treatment and hydrogen peroxide bleaching, all samples exhibited a rough and flaky surface, likely attributed to defibrillation and the removal of non-cellulosic components^26^. Because lignin in the hybridized sample could be oxidized and solubilized using peroxide bleaching, more voids and cellulose fibrils were visible in the structure of the isolated cellulose. Additionally, the combined chemical treatment of all the samples had a stronger peeling effect on the larger-pored fibers, which naturally increased surface area and improved cohesion of the fibers inside the matrix, enhancing physical interlocking to improve bonding and strength^29,35^. Similar observation was reported for extracted cellulose from different lignocellulosic^25,26,35,52^.

Small holes, less surface roughness and flakiness were observed in Sample F (Fig. 9F) indicating that not all the complex matrix was disrupted. This supported the FTIR result which shows that not all hemicellulose and/or lignin were eliminated in the sample. This is because cellulosic biomass structure is protected, stiffened, and impermeable by the lignin, hemicellulose, and waxes and therefore requires optimum isolation condition for the component of interest^53^. Satisfactory flakiness and roughness of the fiber surface was observed for all the remaining samples when the NaOH concentration was increased (Fig. 7a–e). Moreover, the process temperature affects the level of surface roughness, as shown in Fig. 9. While the cellulose in both samples were isolated under 2% NaOH in a comparable way, sample A showed a rougher surface and flakiness when the temperature was elevated from 45 to 95 °C than sample F. This finding suggests that the temperature during the alkali treatment is also important. These conclusions will be validated further by the XRD analysis results.

### XRD analysis

**Figure 10:**
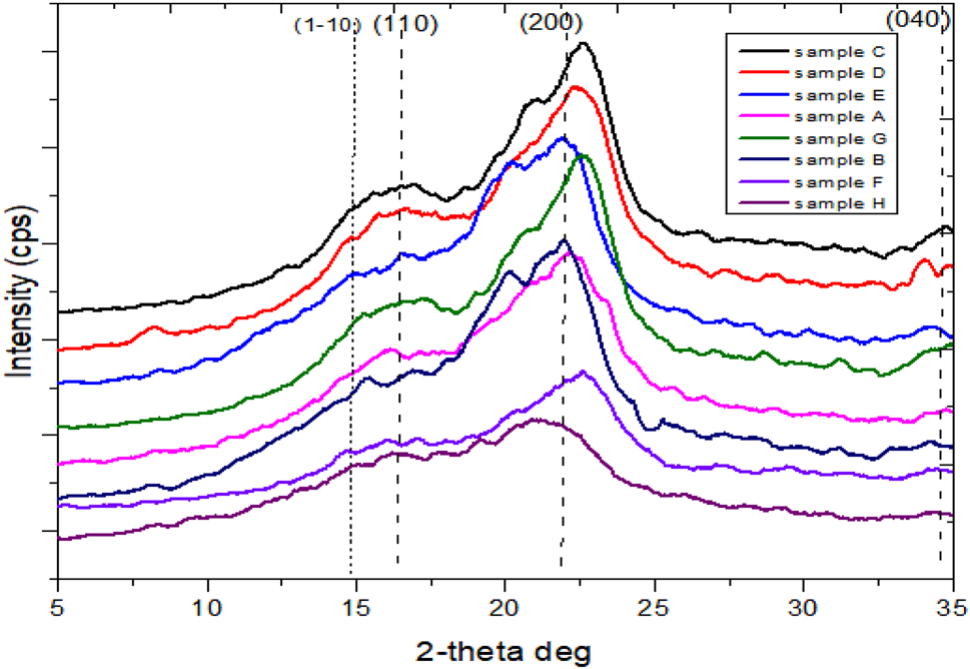
XRD spectra of extract-free sample and synthesized cellulose from different reaction condition.

Figure 10 displays the untreated and isolated cellulose’s diffraction pattern. Table 6 gives each sample’s estimated % C.I and peak height. This technique was adapted to verify whether the non-cellulosic components were successfully confiscated. For the untreated sample as well as the cellulose samples, the primary diffraction peaks are seen at 2θ = 16°, 22° and 34°, which correspond to the crystallographic planes of (1–10), (110), (200) and (040) indicating the cellulose I structure^17,27,48^. The result showed that both the cellulose samples and untreated sample exhibited a crystalline structure denoted by crystalline I. Sample H, the untreated sample has a peak height of 440 cps, corresponding to C.I of 40.7% which is the lowest. The C.I is anticipated to be smaller in the untreated sample than in any of the samples that had undergone the multi-chemical treatment because, some of the non-cellulosic components have solubilized. This is because hemicellulose and lignin are present in the raw sample and both fractions are amorphous in nature^39^. Sample F, which was isolated cellulose at mild conditions (2% NaOH, 45 °C, 10 mL), showed a greater peak intensity of 694 cps correlating to C.I 54.28% than the untreated sample, but it was comparatively lower than the other produced cellulose derived from different reaction conditions. This is because not all the hemicellulose and lignin have solubilized since alkaline concentration and temperature have a significant impact on the isolation process. It is out of the ordinary that alkaline-peroxide treatment increases the number of overall crystalline areas in produced fiber^48^. This is consistent with the findings from the FTIR and SEM examinations of the synthetic cellulose produced under the same reaction condition, which explains why impurities were found in the recovered cellulose treated in mild medium.

**Table 6:** Peak height at corresponding 2 position.

Sample identity	Position at 2θ ≈ 22^o^	Peak height (cps)	Crystallinity index (%)
A	21.22	883	56.78
B	22.07	819	55.93
C	22.54	1217	61.24
D	22.98	925	57.35
E	22.62	1080	59.42
F	22.32	694	54.28
G	22.36	1433	64.13
H	21.30	440	40.87

In contrast, Sample G, which was identified as cellulose isolated at 95 °C and 18% NaOH produced the highest peak intensity of 1433 cps giving rise to the highest C.I of 64.13%. It occurred because of the extraction method's ability to destroy and solubilize the sample's hemicellulose and lignin while successfully isolating the cellulose. As a result, the fiber's overall crystallinity significantly increased because lignin and hemicellulose are amorphous components. The above-mentioned result was in line with the higher C.I attained in the extracted cellulose samples from various agricultural wastes^26,29,35,53^. When it comes to how temperature affects the cellulose isolation process as highlighted, sample A's cellulose was found to have a higher C.I of 56.78% than cellulose separated at a lower temperature (sample F), even though both samples used the same concentration of alkali (2% NaOH). Similarly, sample D has a reasonably lower C.I of 57.35% than sample G despite both celluloses were isolated using the same concentration of NaOH (18%). It is reasonable to argue that the temperature at which cellulose is isolated has just as much significance as the alkali concentration utilized.

Even though a high concentration of NaOH frequently causes crystallite order rearrangement to change the cellulose structure from I to II^35^, the combination treatment left the (1–10) plane without any observable peak. This implies that the crystalline structure of the cellulose was not altered by the reaction conditions, which were not harsh enough. Nevertheless, the difference in the mechanical and physical properties of cellulose can be attributed to the hydrogen bonds in its crystalline sections, which are stronger and more numerous than in its amorphous or non-crystalline portions. For example, crystalline domains have more density and flexibility than non-crystalline ones. Additionally, the disordered portions of the polymer material exhibit flexibility and plasticity, while the ordered regions contribute to the material's stiffness and elasticity. The methods utilized for cellulose extraction affect the degree of crystallinity in cellulose microfibrils.

### Verification of RSM model

Based on the FTIR, XRD and SEM results, sample C demonstrated the high yield of pure cellulose at the lowest possible temperature and moderate concentration of NaOH. Consequently, numerical optimization was conducted using the desirability function within Design Expert Software. The optimization goals were set within the range of extraction conditions observed for sample C, as detailed in Table 7. A total of fifteen distinct solutions were identified, each comprising varying levels of independent variables. The solution yielding the highest desirability rating (0.95) was selected as the optimized condition (Table 7). Subsequently, an experiment was carried out under this optimized condition to validate the model. The results, presented in Table 7, exhibit a reasonable agreement with the predicted values.

**Table 7:** Numerical optimization constraints, optimized conditions, predicted and actual yield at optimized condition.

Name	Goal	Lower limit	Upper limit	Lower weight	Upper weight	Importance	Optimized condition
NaOH (%)	Is in range	5	10	1	1	3	5
Temp. (℃)	Is in range	45	50	1	1	3	45
Vol. H_2_O_2_ (ml)	Is in range	15	20	1	1	3	20
Yield (%)	Maximum	37	46	1	1	3	
Predicted Yield (%)							45.56
Actual Yield (%)							43.9

## Conclusion

The study successfully isolated cellulose from a blend of sugarcane bagasse and corn cob using a multi-step alkaline extraction and hydrogen peroxide bleaching process. Analysis of eight selected agricultural wastes revealed significant cellulose content, ranging from 33 to 41%. FTIR spectroscopic analysis demonstrated similarities in lignocellulosic materials, indicating common functional groups among cellulose, hemicellulose, and lignin. However, sugarcane bagasse and corn cob showed the highest level of cellulose content and thus chosen for isolation. FTIR analysis also revealed that mild reaction conditions resulted in ineffective cellulose isolation due to residual non-cellulosic fractions, while overly harsh conditions led to cellulose decomposition and reduced recovery. The successful elimination of non-cellulosic components was confirmed by FTIR data, while SEM micrographs highlighted the significant impact of alkali treatment temperature on cellulose morphology. XRD results confirmed the phase composition of cellulose. Optimal isolation conditions, considering cellulose yield, extent of crystallinity, surface characteristics, and absence of non-cellulosic components, were determined as 10% NaOH,70 °C, and 20 mL of H_2_O_2_. This research offers a promising method for cellulose isolation from hybridized agricultural waste, facilitating the production of diverse cellulose-derived bioproducts for various industrial applications.

## Data Availability

The data presented in this study are available upon request from the corresponding author.
